# The Advantages of Waist to Height Ratio over the Commonly Used Anthropometric Measurements in Preadolescents and Adolescents: A Case Study from Montenegro

**Published:** 2019-12

**Authors:** Rajko MILASINOVIC, Aleksandar CVOROVIC, Filip KUKIC

**Affiliations:** 1. Faculty of Sport and Physical Education, University of Montenegro, Niksic, Montenegro; 2. Belgrade Football Academy, Belgrade, Serbia; 3. Faculty of Sport and Physical Education, Belgrade University, Belgrade, Serbia

## Dear Editor in Chief

Evaluating and monitoring the anthropo-morphological status of children at school age is a process that must be realized within every serious nationally or internationally driven health system. During the collection of the basic anthropometric measures, a significant data can be collected whose importance can be from individual to the populational or international significance. The most common reasons for collecting these data is to determine the nutritional status of children, or to identify possible health risks due to overweight (including obesity) or malnutrition. However, this is not always that simple, given that the population of one nation can differ significantly, from region to region, and certainly those differences even more fluctuating on international or global level, affected by race, genetic heritage, socio-economic status, environmental factors etc. (1).

Beside from the all aforementioned, in case of school children, situation is even more challenging because of the accelerated growth and biological development especially during adolescence (2). Therefore, the primary objective of this study was to examine which of the simple and commonly used anthropometric measures or indices are the least affected by age or gender of participants. Secondary objective is to recommend possible alternatives/or additional measurements for screening the anthropo-morphological status during the preadolescence and adolescence.

The representative sample of 1478 children (age [Age] =10.97 ±1.39 years (yr.), body height [BH] = 152.75 ±10.73 cm, and body mass [BM] = 45.03 ± 12.43 kg) from Montenegro were evaluated. Further, the participants were stratified by sex: 732 girls (Age = 10.98 ± 1.38 yr., BH = 152.25 ± 10.24 cm, BM = 43.92 ± 11.52 kg) and 746 boys (Age = 10.95 ± 1.41 yr., BH = 153.24 ± 11.18 cm, BM = 46.13 ± 13.19 kg); and by age (9 yr. [Girls vs. Boys, n = 133 vs 147], 10 yr. [n =164 vs. 174], 11 yr. [n = 156 vs. 135], 12 yr. [n = 139 vs. 148] and 13 yr. [n = 140 vs. 142]). Apart from the Age, BH and BM, the waist circumference (WC) was measured, at the level of iliac crest in the mid-axillary line (3) using Gulick tape (North Coast Medical Inc. US) to the nearest cm, and two skinfold thickness measurements (triceps [TrSF] and sub-scapular [SsSF]) using Gima-2 caliper (Gima S.p.A, Italy), and obtained results were recorded to the nearest mm.

The measurements were collected by experienced practitioners from Faculty of Sport and Physical Education, University of Montenegro, during 2017–2018 school year. The body mass index was calculated by formula BMI = BH / BM^2^ and expressed in kg / m^2^. The waist to height ratio (WHtR) was calculated by formula WHtR = WC / BH. Finally, skinfold thickness apart from individual evaluation, were used for creating two more variables SUM2 = TrSF + SsSF, and they were used in regression formula (4) for estimated percent of body fat calculation (PBF). In total 10 variables were generated for further statistical analysis Age, BH, BM, BMI, WC, WHtR, TrSF, SsSF, SUM2 and PBF. Beside the mean and standard deviation [SD]), the Pearson’s product correlations were used to evaluate association between variables related to age, stratified by gender and in total sample of participants.

The data analyses were conducted using SPSS version 20 (Chicago, IL., USA), with significance level set for values of *P*<0.05 a priori. The basic descriptive characteristics of participants are shown in Table 1, while correlations of age with other variables are presented in Table 2.

**Table 1:** Participants characteristics

***Gender (N)***	***Descriptive Statistic***	***Age (year)***	***BH (cm)***	***BM (kg)***	***BMI (kg/m^2^)***	***WC (cm)***	***WHtR (cm/cm)***	***TrSF (mm)***	***SsSF (mm)***	***SUM2 (mm)***	***PBF (%)***
Girls	Mean	10.98	152.25	43.92	18.69	67.53	0.44	15.13	11.11	26.23	24.50
(N=732)	SD	1.38	10.24	11.51	3.37	9.40	0.05	5.50	5.92	10.75	10.49
Boys	Mean	10.95	153.24	46.13	19.33	69.66	0.45	14.72	10.65	25.36	23.99
(N=746)	SD	1.41	11.18	13.19	3.71	10.92	0.06	6.27	6.80	12.45	12.14
Total	Mean	10.97	152.75	45.03	19.01	68.61	0.45	14.92	10.88	25.79	24.25
(N=1478)	SD	1.39	10.73	12.43	3.56	10.25	0.06	5.90	6.39	11.64	11.35

**Table 2:** Age related correlations by gender and for total sample

***Gender***	***BH***	***BM***	***BMI***	***WC***	***WHtR***	***TrSF***	***SsSF***	***SUM2***	***PBF***
Girls	0.747^**^	0.590^**^	0.315^**^	0.346^**^	−0.014	0.080^*^	0.172^**^	0.135^**^	0.129^**^
Boys	0.754^**^	0.559^**^	0.258^**^	0.340^**^	−0.008	−0.025	0.089^*^	0.036	0.036
Total	0.749^**^	0.569^**^	0.282^**^	0.338^**^	−0.012	0.023	0.127^**^	0.081^**^	0.078^**^

** Correlation is significant at the 0.01 level (2-tailed)

* Correlation is significant at the 0.05 level (2-tailed)

The results suggest that the WHtR was the only variable that was not affected by age or gender of this representative sample of Montenegrin children, regardless of sex and age.

These findings qualify WHtR as the simplest, but yet, very reliable method for estimation of health risks from overweight and obesity in children of that age (5). The application of WHtR potentially can be very useful in countries or regions without developed national references for BMI-for-age and gender, or some other references, such as skinfold thickness or WC.
